# Heavy commercial vehicles' mobility: Dataset of trucks' anonymized recorded driving and operation (DT-CARGO)

**DOI:** 10.1016/j.dib.2023.109246

**Published:** 2023-05-18

**Authors:** Georg Balke, Lennart Adenaw

**Affiliations:** Technical University of Munich, TUM School of Engineering and Design, Chair of Automotive Technology, Boltzmannstr. 15, 85748 Garching, Germany

**Keywords:** Road freight transport, Truck, GNSS, Mobility data, Fleet data, Logistics

## Abstract

During a period of 7 months, 54 class N3 trucks from 4 fleets of German fleet operators were equipped with high resolution GPS data loggers. A total of 1.26 million km of driving data has been recorded and constitutes one of the most comprehensive open datasets to date for high-resolution data of heavy commercial vehicles. This dataset provides metadata of recorded tracks as well as high-resolution time series data of the vehicle speed. Its applications include simulation of electrification for heavy commercial vehicles, modeling logistics processes or driving cycle construction.

**Specifications Table**

**Table:** 

Subject	Automotive Engineering
Specific subject area	GNSS Recordings, Truck Electrification, Transport Management
Type of data	Mobility Data Table
How the data were acquired	The data were collected using GPS data loggers developed at the Technical University of Munich. The loggers were installed in 54 heavy-duty trucks to record movement data. In order to anonymize the dataset to make it publicly available, GPS coordinates were removed and replaced by track-wise computed information (e.g. track distance) and semantic geographical classification of locations (e.g. service areas) using secondary data from Open Street Maps and proprietary information available from the fleet operators.
Data format	Raw data Analyzed Processed
Description of data collection	54 data loggers were installed in heavy-duty trucks operated by 4 companies between fall 2021 and spring 2022 to continuously measure position and speed with a frequency of 10 Hz. The data were retrieved from the loggers and fed into a PostgreSQL database. Using this database, the recorded GPS traces were pre-processed to create the anonymized and augmented csv-exports published herein.
Data source location	Institution: Technical University of Munich, TUM School of Engineering and Design, Chair of Automotive Technology City/Town/Region: D-85748 Garching Country: Germany Longitude (collected data) between 3.7768° E and 14.9671° E Latitude (collected data) between 45.5992° N and 54.4140° N
Data accessibility	Repository name: Zenodo Data identification number: 10.5281/zenodo.7599687 Direct URL to data: 10.5281/zenodo.7599687 Instructions for accessing these data: The repository contains a set of compressed csv files. A concise description of their contents and the code used for visualization is provided under https://github.com/TUMFTM/dt-cargo.

## Value of the Data

• To the best of the authors’ knowledge, this is one of the most comprehensive datasets of heavy-duty truck mobility publicly available. It offers a sample of heavy commercial vehicle's movements in the form of a trip logbook comprising both data on trip distance, duration and speed as well as semantic information on trip destinations. Additionally, high-resolution speed profiles and vehicle type information are provided.
• Transportation and automotive engineers, researchers, and public authorities may find the dataset useful especially in the context of electrification for benchmarking heavy-duty truck operation cycles, as it includes detailed information on speed profiles, mileages, operation times, and locations.
• While the sample size may be limited in comparison to the hundreds of thousands of trucks on European roads, the dataset provides valuable and very detailed vehicle-based insights into prototypical operation cycles of heavy-duty trucks that may be used to develop electric vehicle concepts tailored to real usage patterns and for general transportation planning.

## 1 Objective

An important step in the electrification of commercial vehicles is the understanding of usage pattern and requirements. This dataset was created to provide a detailed view of commercial vehicle utilization and can be employed to develop optimized electric commercial vehicle concepts. The speed-profiles can provide an input for longitudinal dynamics simulations, while spatial context information can be evaluated for possible charging infrastructure.

## 2 Data Description

This article refers to three published datasets. A characterization of the recorded vehicle fleets (*fleet.csv*), the recorded vehicle tracks (*tracks.csv*), and speed profiles for each track (*{track_id}.csv*). The data is provided online [1]. In order to provide a clearer picture of the contents, short tracks (<=1000 m) are excluded from the following assessments. They make up 82,024 of the 101,826 recordings and mostly contain local shunting and parking operations. Table 1 contains the recorded distances and durations per vehicle fleet.

**Table 1:** Global information on the recorded fleets, tracks shorter than 1000 m are excluded.

Fleet	Vehicles	Recorded distance / 1000 km	Recorded time / h
1	18	368.43	7494.21
2	5	137.88	3149.70
3	12	170.99	5118.66
4	18	583.61	9227.61

In total, 1260,908,125 km were recorded from four different fleets during the experiment. Fleet four accounts for the largest distance share (46 %). The total recording time of 24,990 h is distributed more evenly, with fleet four providing the largest share at 37 %. The median vehicle recorded 26,780 km.

### 2.1 {track_id}.csv

For each recorded track, the time series of speed and measurement precision is provided in an individual file named after its corresponding track ({track_id}.csv), placed in the folder named after the corresponding vehicle id. Table 2 shows the structure of these files.

**Table 2:** Data structure of the {track_id}.csv.

Column	Data Type	Unit	Description
Epoch	float	s	Unix timestamp of measurement in time zone “Europe/Berlin”
Speed	float	m/s	Speed at measurement in m/s
Hdop	float	m	Horizontal degree of precision during recording [2]

**Fig. 1:**
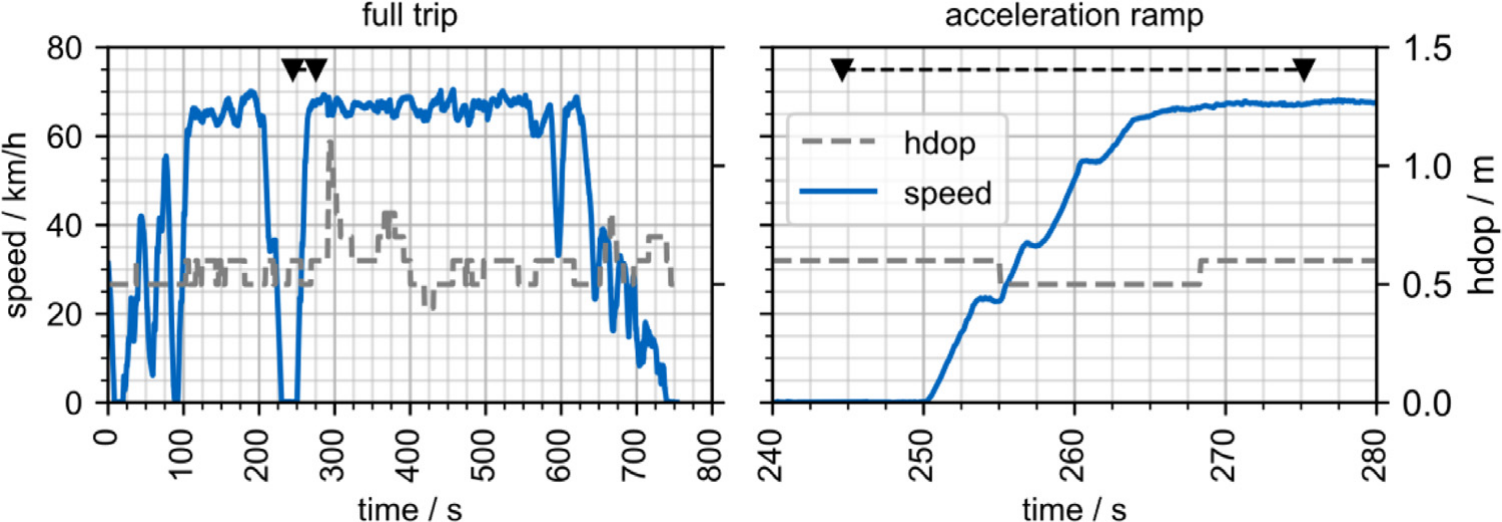
Exemplary speed profile and horizontal degree of precision. The visualization is based on track 10 of vehicle 1. Left: Overview; Right: Acceleration ramp detail.

The data is provided with a frequency of 10 Hz. Fig. 1 illustrates an example recording of 753 s with an average speed of 48.9 km/h. The track consists of 3 micro trips, separated by a 4 s-stop at 88 s and a 20 s-stop at 250 s. The acceleration ramp at the beginning of the third micro trip is displayed in detail (Fig. 1), indicating the resolution of the data. During the 20 s of acceleration, four clear saddle points, resulting from gear changes, can be observed.

### 2.2 fleet.csv

The *fleet.csv* file contains an overview of the vehicles used in the fleet test. Their weight classes and axle configurations are provided. The axle configuration classes of the Federal Highway Research Institute (Bundesamt für Straßenwesen, BaSt) are utilized [3]. Table 3 documents the file structure.

**Table 3:** Data structure of the fleet.csv.

Column	Data Type	Unit	Description
vehicle_id	Int	–	Unique serial id of each vehicle
fleet_test_id	Int	–	Unique serial id of the fleet the vehicle belongs to
gross_vehicle_weight	Int	kg	Gross Vehicle Weight Rating (without trailer)
total_mass_with_trailer	Int	kg	Gross Combination Weight Rating (with trailer, equals gross_vehicle_weight if no trailer can be attached)
axle_class	Int	–	Vehicle class according to [3]

### 2.3 tracks.csv

The *tracks.csv* provides an overview of the recorded tracks with meta-information and is described in Table 4. A track is a single recording, started and stopped according to the criteria listed in Section 3.1. A tour is a chain of tracks that starts and ends at the home base.

**Table 4:** Data structure of the tracks.csv.

Column	Data Type	Unit	Description
track_id	Int	–	Unique serial id of each recorded track (ordered by vehicle_id and start_time)
vehicle_id	Int	–	Unique serial id of each vehicle
tour_id	Int	–	Serial id of each tour, assigned to 1..N tracks
start_time	Timestamptz	–	Start time of the recording with time zone at time of recording
stop_time	Timestamptz	–	Stop time of the recording with time zone at time of recording
distance	Float	m	Distance driven during track
track_gap	Float	m	Distance gap to following track
avg_speed	Float	m/s	Average speed
max_speed	float	m/s	Maximum speed within track
n_signal_loss	float	–	Number of signal loss events during recording
d_signal_loss	float	m	Distance covered during signal losses
r_signal_loss	float	–	Ratio of signal loss distance to recorded distance
avg_hdop	float	m	Average horizontal degree of precision during recording
home_base	bool	–	End of recording is at home base of fleet operator
long_haul	bool	–	End of recording is more than 150 km away from home bases
rest_area	bool	–	End of recording is at an unserviced rest area
service_area_fuel	bool	–	End of recording is at a service area
industrial_area	bool	–	End of recording is in an industrial area
cid	int	–	Cluster id of last location in recording, described in Section 2.

### 2.4 Time of Recording

**Fig. 2:**
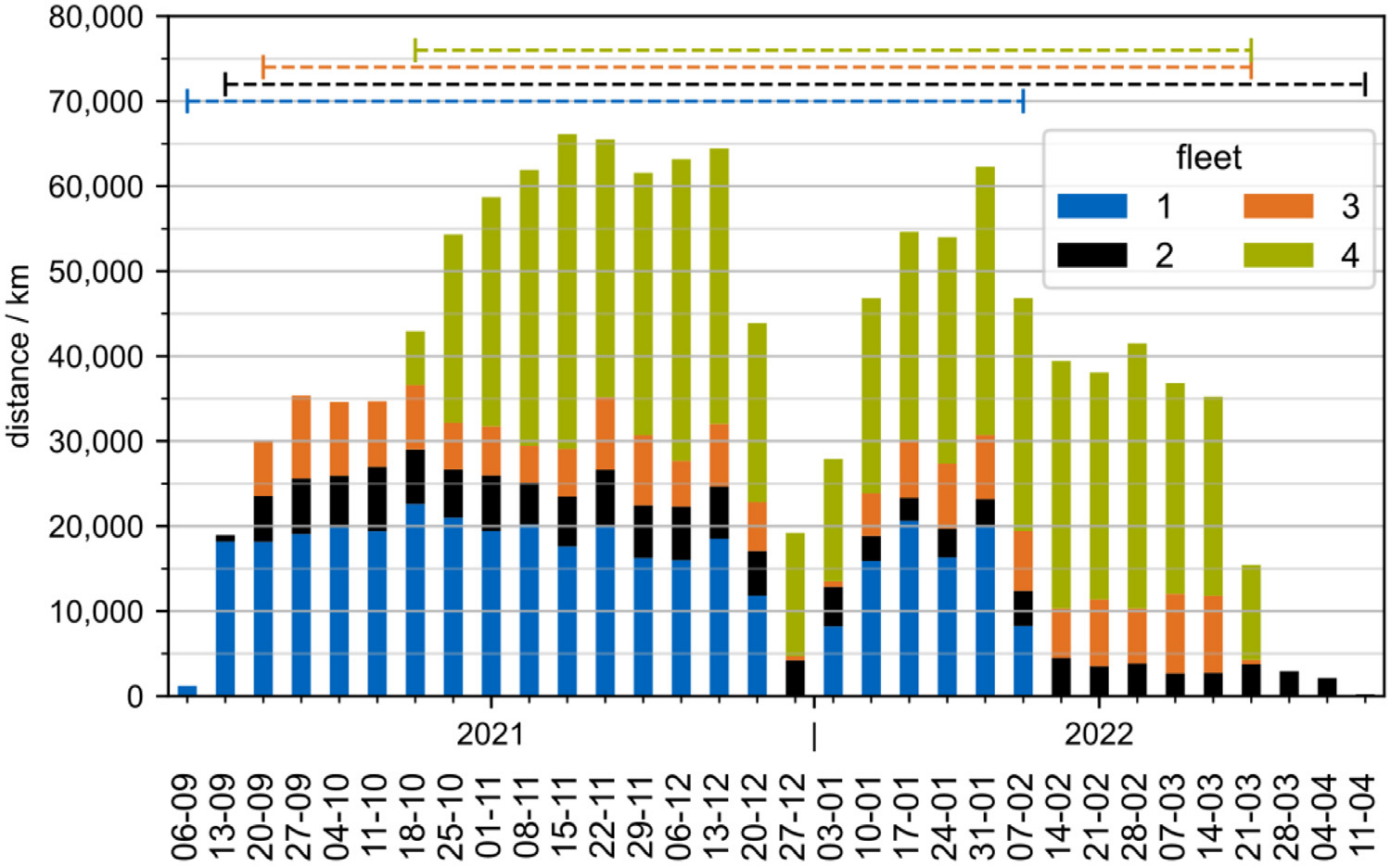
Recorded distance per calendar week and fleet. Horizontal lines indicate the time between the first and last recording per fleet. The visualization is based on tracks.csv and fleet.csv. Tracks shorter than 1000 m distance are excluded.

The recordings took place between September 7 2021, and April 11 2022,. The first fleet to record was fleet one, the other fleets followed in the order of their fleet ids. A ramp-up and phase-out can be observed for all fleets, as data loggers were installed and removed gradually (Fig. 2). Between December 27 and January 10, a decline in recorded kilometers can be observed for all fleets. In the week following December 27, the total mileage is only 30.2% of a median November week.

### 2.5 Track Distance and Duration

**Fig. 3:**
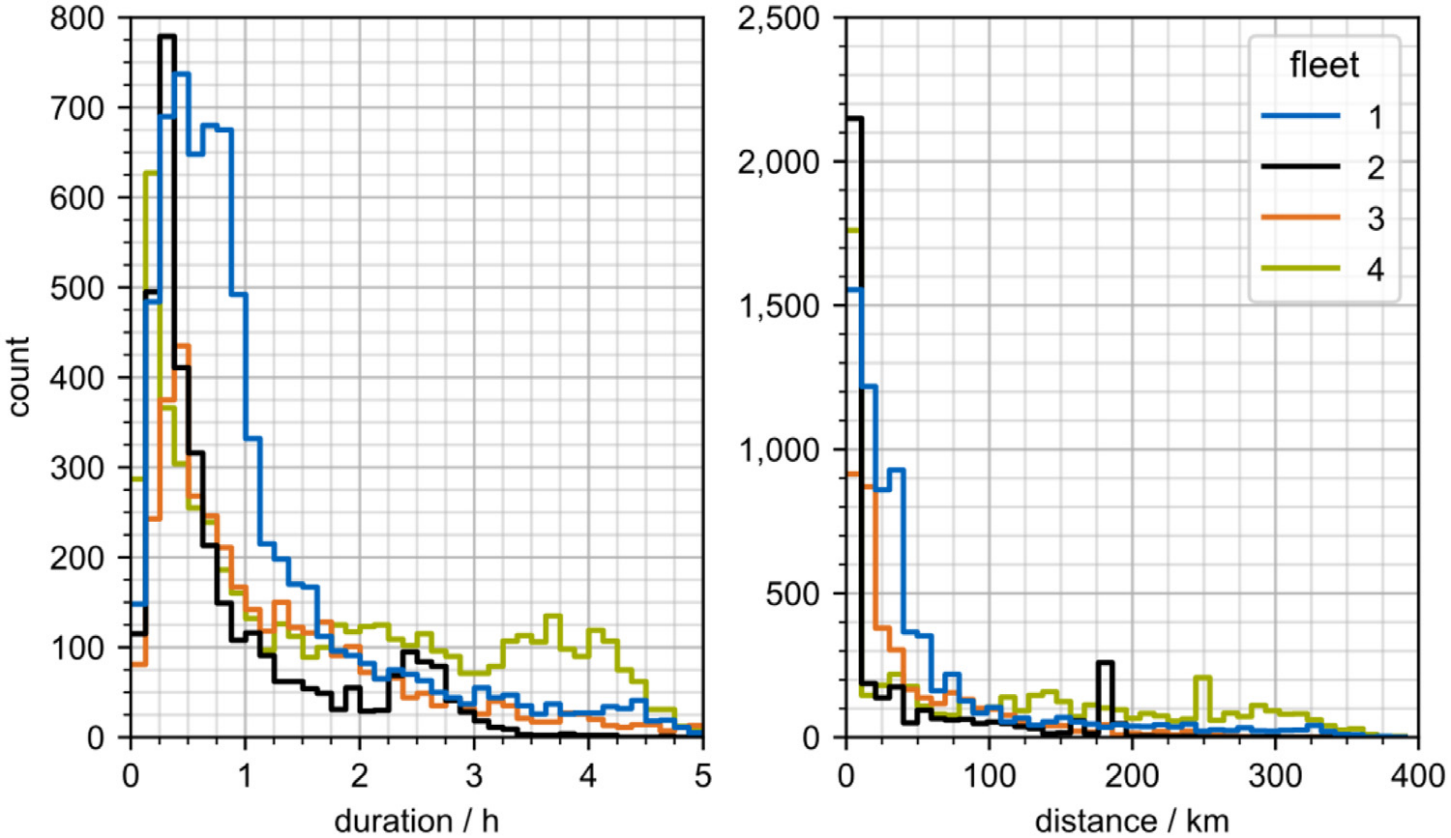
Histogram of distance and duration of tracks. The figures are limited to a maximum of 5 h and 400 km. Of the recorded tracks over 1000 m, 0.6 % exceed 5 h and none are longer than 400 km. The visualization is based on tracks.csv and fleet.csv. Tracks under 1000 m distance are excluded.

The most frequent track durations of all fleets are below one hour (Fig. 3). However, fleets two and four feature local peaks at 2.5 h and 3.7 h respectively. The median track duration for fleet two and fleet four ranges from 0.51 h to 1.31 h respectively.

Similarly, the majority of track distances for all fleets falls below 100 km. Long-haul tracks exceeding 150 km are present in all fleets, with fleet four displaying the highest proportion of tracks above 150 km. The median track distance for fleet two and fleet four ranges from 4.4 km to 73.1 km respectively.

The data indicates that while the average speed across all fleets is 38.95 km/h, tractors (axle class 98) exhibit a higher average speed of 48.60 km/h. A national-level survey finds, that tractors have an average speed of 51.90 km/h [4, A22.1] while the average fixed body truck above 3.5 t is slightly slower at 46.51 km/h [4, A21.1]. Considering the average track distance, representative figures show a high variance between fixed body trucks at 15.04 km [4, A21.1] and tractors at 79.11 km [4, A22.1]. The provided dataset's values are between the two values at 63.85 km.

### 2.6 Rest Time Distribution

**Fig. 4:**
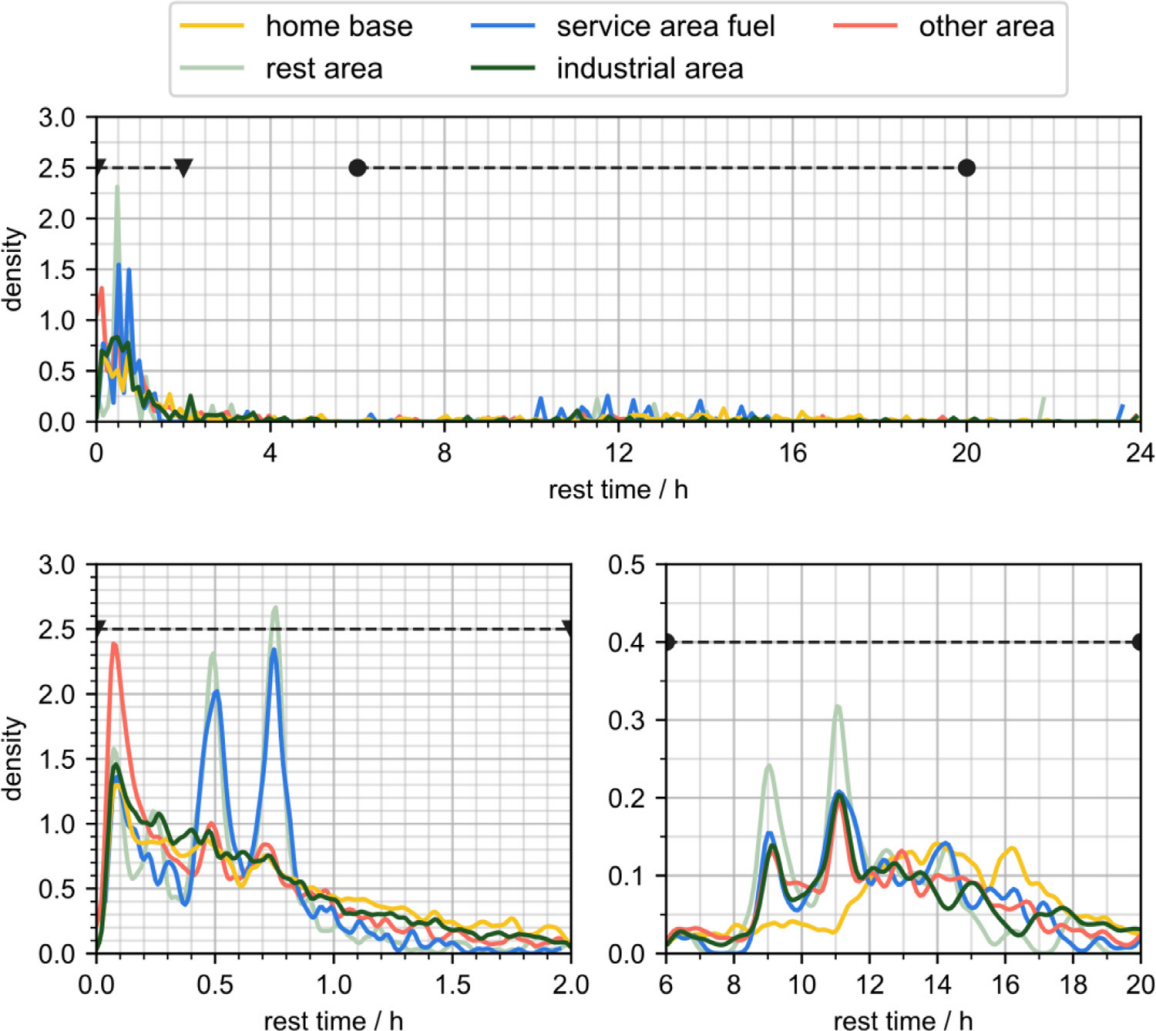
Kernel density estimation of the recorded rest period. The visualization is based on tracks.csv and fleet.csv and tracks under 1000 m distance are excluded.

Fig. 4 displays the rest times of the vehicles at different locations using a kernel density estimation. Following Scott's rule [5], the kernel density estimation uses a Gaussian kernel with bandwidth 0.2 · n^(−1/5) for the n samples within each location type. Two key intervals can be identified in which rest times frequently occur: The highest density can be observed for durations shorter than two hours. The second interval spans from 6 to 20 h. Both intervals are additionally displayed in detail, to reveal their characteristics.

In the first interval, containing rest times shorter than two hours, stops at service areas and rest areas are different from other rest locations. Both exhibit density peaks at 30 min and 45 min. In contrast, stops at home bases and foreign industrial areas decline in probability as duration increases. Stops at unidentified areas have a high probability of being shorter than half an hour, peaking at 6 min.

The second interval which contains longer stops, includes peaks at 9 and 11 h observable for the locations rest area, service area, other area, and industrial area. Only stops at the home base do not display this pattern, having the highest relative probability at 14 hours.

### 2.7 Clusters

Frequent destinations are described in the dataset through clusters. Analyzing the distribution of the stops among the 740 identified clusters, it can be observed, that in more than half of the cases, a small cluster with less than 400 visits was the track destination. The largest cluster, cluster_id 0 was visited 1915 times and can be identified as the home location of fleet 1. A total of 1418 outlier tracks were not assigned to a cluster. Table 5 provides an overview of the visiting vehicles of clusters at different sites. It can be observed, that home locations are the most frequented type of cluster, counting visits and unique vehicles visiting. Among the other identifiable areas, industrial area clusters are the second most visited cluster type with 23.81 visits per cluster on average, while service areas are visited by more distinct vehicles at 4.81 on average.

**Table 5:** Statistics of visits at identified clusters. A track of over 1000 m ending at an identified cluster is counted as a visit.

		home base	industrial area	rest area	service area	other area
**visits**	**lower quartile**	417.50	3.00	4.00	4.00	2.00
	**Median**	780.00	7.00	5.00	6.00	5.00
	**Mean**	988.14	23.81	6.51	9.35	16.45
	**upper quartile**	1552.50	16.00	8.00	9.00	9.00

**unique** **vehicles**	**lower quartile**	10.50	2.00	2.75	3.00	1.00
	**Median**	12.00	3.00	3.50	4.00	2.00
	**Mean**	12.29	4.36	3.78	4.81	3.25
	**upper quartile**	15.00	5.00	5.00	6.00	4.00

### 2.8 Fleet occupation

**Fig. 5:**
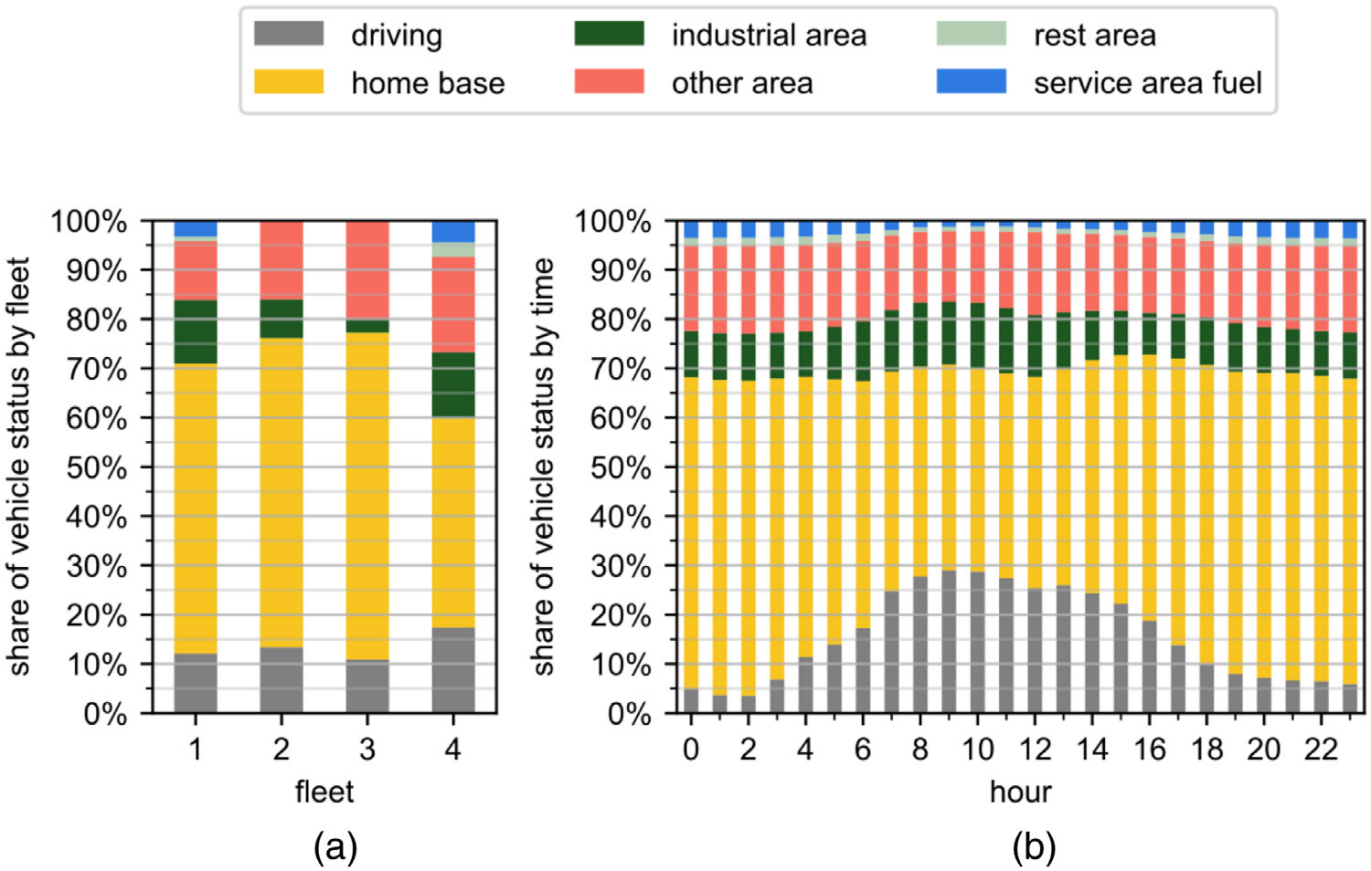
Occupation of the vehicles during the research period. (a) Vehicle status grouped by fleet, (b) vehicle status by hour of day. The evaluated interval includes working and non-working days. The visualization is based on tracks.csv and fleet.csv and tracks under 1000 m distance are excluded.

In Fig. 5, two dependencies considering the occupation of vehicles are displayed: The total time spent resting at different locations or driving is displayed in Fig. 5a. The occupation of all fleets aggregated over the course of 24 hours is provided in Fig. 5b.

For all fleets, dwelling at the home base is the most common occupation. Driving only accounts for 10 % to 17 % of the investigated period, depending on the fleet. Representative figures show, that tractors are driving 21.4% of the week, while fixed body trucks above 3.5t only drive for 10.03% of the week [4, A21.1, A22.1]. While fleets one and four spend 4.2 % to 7.4 % of the time at rest and service areas, fleets two and three only spend 0.22 % and 0.18 % there.

When examining the intra-day variance, driving and dwelling at an industrial area are more prevalent during the day, while dwelling at rest areas, service areas, and the home base are more frequent at night. The highest proportion of vehicles on the road is reached between 9 a.m. and 10 a.m., while the most vehicles begin driving between 6 a.m. and 7 a.m. For unidentified areas, no clear trend can be observed.

### 2.9 Data quality

**Fig. 6:**
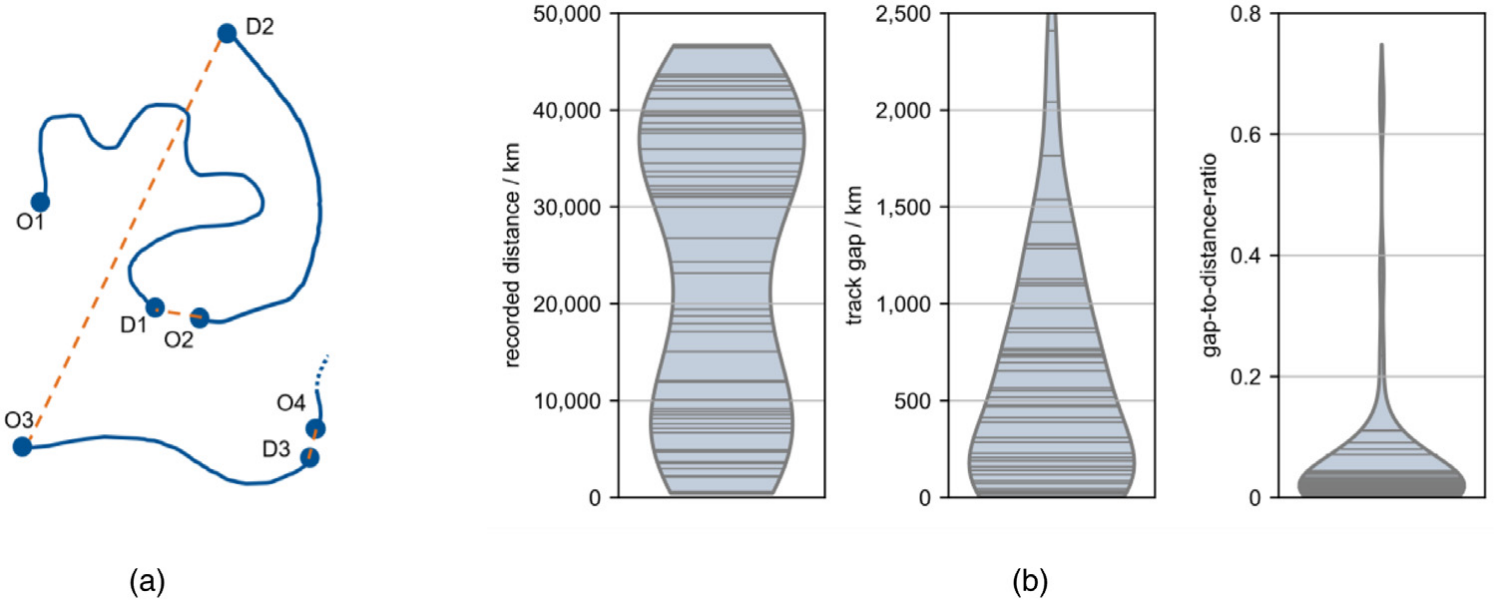
(a) Schematic depiction of the tracks (solid blue) and track gaps (dashed orange) between origin (O) and destination (D) of tracks 1 - 3. The gap distance is calculated as the aerial distance from the track's destination to the following track origin. (b) Violin plots of the total recorded distance, total track gap, and gap-to-distance ratio. Each line represents one vehicle.

In order to assess the completeness of data, unrecorded distances can be evaluated. In some cases, a recording stopped during driving, in other cases whole tracks could not be recorded. Main reasons for missing measurements are technical issues in the software, hardware problems of sensors and suboptimal track recognition. To estimate the data quality of the recordings, an estimation of completeness is carried out. The process is visualized in Fig. 6a.

The recorded distance of a vehicle $d_{rec}$ is calculated as the sum of its track lengths (1). The sum of its track gaps $d_{gap}\;$is calculated as the line-of-flight distances between the recorded tracks (2). The ratio r of unrecorded gaps to recorded distance is then evaluated per vehicle (3) and visualized in Fig. 6b.

(1) $$d_{rec}=\sum_{i}l_{track,i}$$

(2) $$d_{gap}=\sum_{i}d\left(O_{i+1}D_{i}\right)$$

(3) $$r=\;\frac{d_{gap}}{d_{rec}}\;$$

The distance gap between the end of the track and the start of the succeeding track is provided as column track_gap in tracks.csv and visualized in Fig. 6b. It can be observed that the gap-to-distance ratio is smaller than 0.2 for all but for two vehicles.

## 3 Experimental Design, Materials and Methods

**Fig. 7:**
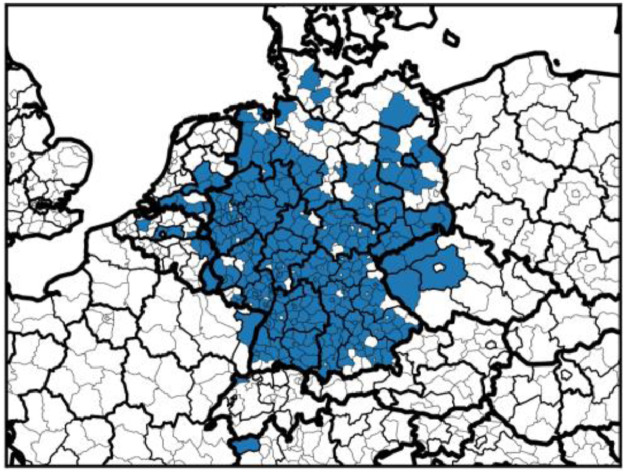
The NUTS-3 regions that at least one track ended in. Dense coverage can be observed in southern and western Germany. Countries include Austria, Belgium, Czech Republic, France, Germany, Italy, Luxemburg, Netherlands, and Switzerland.

During a period of seven months, data loggers were installed in 54 trucks of four fleets. These fleets of heavy commercial vehicles are operated by companies that take part in the “NEFTON” research project (grant 01MV21004A) and were selected in order to represent a wide variety of applications of trucks. Fig. 7 provides a spatial overview of the research area.

**Fig. 8:**
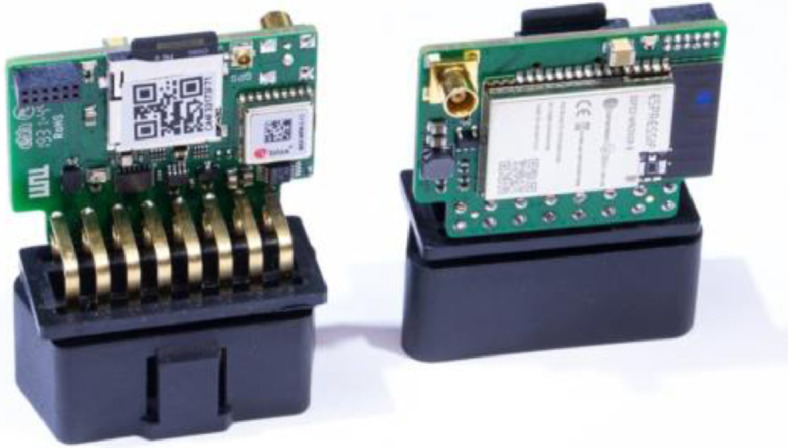
The data logger developed at the Institute of Automotive Engineering, featuring an ESP-32 microcrontroller, a MAX-M8W GNSS module by ublox and an external GNSS Antenna.

The data loggers (Fig. 8) serve the purpose of providing high-quality data in a reliable manner. They are equipped with a 32GB SD card and able to operate with low energy consumption and no required online connection for several thousand kilometers. The data published was obtained through the IMU and voltage sensors as well as the GPS module of the data logger, while the diagnostic connector was solely used for energy supply.

### 3.1 Track Recognition

Tracks were detected during operation by the data logger. The information was derived from the recorded GPS data based on parametrized start and stop conditions. These binary stop conditions$\;s_{i}$, evaluated every 100 ms, indicate a possible stop of the vehicle and are described in Table 6. In order to improve track recognition and decrease sensitivity against micro trips, the stop conditions are combined to a unitless composed stop score $s_{stop}$. The composed stop score exceeding the threshold $s_{stop,thr}$ for a time greater than $t_{stop,1}$ leads to a stop mark being set. The calculation of $s_{stop}$ is described in (2). If the stop conditions are violated again within $t_{stop,2}$, it is assumed that the stop was brief and the track is continued. The parameters$\;p_{i}\;$used during data collection are listed in Table 7. The conditions and parameters were tuned during previous research projects in order to avoid missing movements of the vehicle, as false positives can easily be filtered out when post-processing the data. Equation 2 can be summarized as that in most cases a combination of two stop conditions ends a track, while voltage drops only stop a track in combination with a generally low system voltage.

(2′) $$s_{stop}=p_{IMU}\;s_{IMU}+\;p_{VOL}\;s_{VOL}+\;p_{GPS}\;s_{GPS}+\;p_{SPD}\;s_{SPD}+\;p_{VCH}\;s_{VCH}$$

**Table 6:** Stop conditions for tracks.

Quantity	Calculation	Explanation
$s_{IMU}$	$∥\alpha ∥<1.3_{s^{2}}^{m}or∥\alpha ∥<1.5_{s^{2}}^{rad}$	**α**: Translational and rotational acceleration high-pass filtered
$s_{VOL}$	U<27.6V	U: Voltage of vehicle's electrical system
$s_{GPS}$	Less than 100 m traveled atv<1m/s	*ν* : vehicle speed Slow movement at end of tracks with length of at least 800m
$s_{SPD}$	v<6m/s	Stop condition unset if speed rises above 6 m/s again
$s_{VCH}$	ΔU<−0.5V	ΔU: Voltage drop due to alternator being shut off

**Table 7:** Parameters for stop score calculation.

Parameter	Value	Unit
$p_{IMU}$	15	–
$p_{VOL}$	25	–
$p_{GPS}$	15	–
$p_{SPD}$	15	–
$p_{VCH}$	10	–
$s_{stop,thr}$	26	–
$t_{stop,1}$	10	s
$t_{stop,2}$	20	s

In contrast, tracks are started when $s_{IMU}$ and $s_{VOL}$ are both violated within $t_{stop,1}$.

### 3.2 Metadata Calculation

All track-specific metadata is calculated during post-processing. In order to compute this metadata from the location and speed measurements, the data processing pipeline presented in [6] is utilized.

### 3.3 Track-Specific Metadata

In order to filter outliers, all GPS points that violate the following two quality criteria are excluded from the calculation

• horizontal degree of precision (hdop) smaller than 50 m (Q.1)
• GPS speed between 1.388 m/s and 75 m/s (Q.2)

The distance between two locations is then computed according to the Haversine-Formula and accumulated to calculate track distance (distance). The time difference between the first and last measured point of a track is used as the track duration (duration).

To calculate the average speed (avg_speed) of each track, the track distance is divided by the track duration. It is thus a quantity derived from the location measurements.

Contrary to this, the maximum speed (max_speed) is the maximum instantaneous recorded velocity measured by the GPS module. Internally, it evaluates Doppler measurements of the GNSS signals and thus does not have to rely on differential velocity calculation.

### 3.4 Spatial Features

To provide spatial context information on the dataset and to retain the participants anonymity, GPS track destinations are matched to and replaced by meaningful area descriptions based on Open Street Maps (OSM) data. Each track end is assigned five boolean labels described in Table 8 based on its last measured location being within a tolerance zone around the respective OSM features. A detailed description of the labeling guidelines within Open Street Maps can be found on [7].

**Table 8:** OSM-based location labels for track ends.

Column	Source	Description	Tolerance
service_area_fuel	OSM ways and relations with tag highway = service_area	Service areas along the motorway, generally offering a fuel station and other amenities like a restaurant and toilets	200 m
rest_area	OSM ways and relations with tag highway = rest_area	Unserviced areas along motorways and highways.	200 m
industrial_area	OSM ways and relations with tag landuse = industrial	Areas used for industrial purposes.	200 m
home_base	Manually added locations of fleet operators	Sites used for loading, unloading, parking etc.	according to site size
long_haul	Manually added locations of freight forwarders	Aerial distance to closest home base > 150 km	–

For evaluation purposes and visualization in Section 1, the tags home_base, service_area_fuel, rest_area and industrial area are prioritized in that order, e.g. a location that is a home base in an industrial area is classified as a home base. The tag long_haul is based on the fleet operators’ company premises.

The trucks re-visit certain destinations. A DBSCAN clustering is applied using an existing implementation within the PostGIS framework [8] to provide information on the cyclicity of movement, using the parameter listed in Table 9. The parameter *minpts* describes the minimal number of tracks that have to end at a similar location to constitute a cluster, while *ε* is the search radius of the DBSCAN algorithm. In order to calculate the distances between the track destination, a euclidian metric is utilized internally. Thus, the track destinations are reprojected to the Spherical Mercator projection (EPSG:3857) in order to provide a continuous coordinate system throughout the survey area. The resulting cluster id (cluster_id) is assigned to each track, representing the cluster the track destination belongs to, or −1 if no cluster could be assigned (outlier/noise).

**Table 9:** Parameters of the DBSCAN algorithm used to cluster reoccurring track destinations.

Parameter	Value
ε	1000 m
*minpts*	5
Projection	EPSG:3857

In order to construct activity chains from tracks, the tours are reconstructed. A tour, in individual mobility, describes a chain of movements that begins and ends at home. In this context each chain of tracks starting and ending at a home base is considered a tour.

Using this definition, a unique tour_id is generated and assigned to all tracks belonging to a tour. The conditions for the start and end of a tour are as follows:

• consecutive tracks without a stop at a home base constitute a tour
• a tour ends when a home base is reached
• the next tour starts immediately, including all tracks inside the home base
• a track between two home bases with different cluster IDs is considered a tour.

### 3.5 Quality Measures

A set of quality measures is calculated and provided for each track.

If a location measurement fails the two quality criteria (Q.1) and (Q.2) or if no location is recorded for at least three median recording periods, a signal loss counter is increased by 1. The total number of signal loss events during a track is then provided as n_signal_loss. The line-of-flight distance between the last valid measurement before a signal loss event, and the first valid measurement after the signal loss is calculated and added up per track (d_signal_loss). The ratio of signal loss distance and track distance is provided for each track as r_signal_loss.

The horizontal degree of precision is saved during each location measurement and averaged for each track, yielding avg_hdop. It is an estimate of the standard deviation of the location [2].

## CRediT authorship contribution statement

**Georg Balke:** Conceptualization, Methodology, Software, Formal analysis, Investigation, Data curation, Writing – original draft, Writing – review & editing, Visualization. **Lennart Adenaw:** Software, Formal analysis, Visualization, Writing – original draft, Writing – review & editing, Supervision.

## Declaration of Competing Interest

The authors declare that they have no known competing financial interests or personal relationships that could have appeared to influence the work reported in this paper.

## Data Availability

Dataset of Trucks' Anonymized Recorded Driving and Operation (Original data) (Zenodo).

## References

[1] Georg Balke, & Lennart Adenaw. (2023). Dataset of Trucks' Anonymized Recorded Driving and Operation (1.0) [Data set]. Zenodo. doi:10.5281/zenodo.7599687. PMC10293978 37383791

[2] u-blox, “MAX-M8 series u-blox M8 concurrent GNSS modules Data Sheet”, UBX-15031506-R05 [Revised May 2019].

[3] Bundesanstalt für Straßenwesen, „Datensatzformat der Achslast-Jahresauswertungen (ALJA)“, 2018. Accessed 22 January 2023 [Online]. Available: https://www.bast.de/DE/Statistik/Achslast/Daten/Daten-Beschreibung.pdf.

[4] M. Wermuth et al., “Kraftfahrzeugverkehr in Deutschland 2010 (KiD 2010) Schlussbericht”, Brunswick 2012.

[5] Scott D.W. (1992).

[6] Wittmann M. (2017). 2017 IEEE 20th International Conference on Intelligent Transportation Systems (ITSC), Yokohama, Japan.

[7] OpenStreetMap Contributors: “OpenStreetMap Wiki”, Accessed 22 January 2023 [Online]. Available: https://wiki.openstreetmap.org/.

[8] POSTGIS: “ST_ClusterDBSCAN”, Accessed 22 January 2023 [Online]. Available: https://postgis.net/docs/ST_ClusterDBSCAN.html.

